# Pancreatic carcinoma in situ with focal pancreatic parenchymal atrophy diagnosis using serial pancreatic juice aspiration cytological examination via the minor papilla

**DOI:** 10.1055/a-2587-8921

**Published:** 2025-05-09

**Authors:** Tatsunori Satoh, Shinya Kawaguchi, Hideyuki Kanemoto, Kazuya Ohno

**Affiliations:** 1 26389 Department of Gastroenterology, Shizuoka General Hospital, Shizuoka, Japan; 2 26389 Department of Surgery, Shizuoka General Hospital, Shizuoka, Japan

To improve pancreatic ductal adenocarcinoma prognosis, early diagnosis is crucial. Assessment by serial pancreatic juice aspiration cytologic examination (SPACE) after placing a naso-pancreatic drainage (NPD) tube has proved effective (including pancreatic carcinomas in situ [CIS]) 1 2 3 . This procedure is applicable for patients with indirect pancreatic carcinoma indications, such as dilation of the main pancreatic duct (MPD) or focal pancreatic parenchymal atrophy (FPPA). The standard practice is placing an NPD tube in the MPD through the main papilla; yet, this is not always possible. We previously demonstrated the feasibility of endoscopic retrograde pancreatography (ERP) for pancreatic divisum via the minor papilla (MP) 4 . Here, we report a case of CIS with FPPA diagnosed using SPACE via the MP ( Video 1 ).

NPD tube was successfully placed on the MPD via the minor papilla for serial pancreatic juice aspiration cytologic examination (instead of the standard main papilla).Video 1

An 81-year-old man was referred to our department upon FPPA detection on computed tomography performed for pulmonary disease assessment. Multiple standard imaging modalities failed to reveal pancreatic mass ( Fig. 1 ). Thus, CIS was suspected, and SPACE was planned. First, major papilla cannulation for MPD was attempted; however, cannulation was unsuccessful. Thereon, the orifice of MP could be detected and a guidewire was advanced upto the MPD via MP. Fluorography revealed a slight stenosis of the MPD, consistent with FPPA. As deep cannulation was difficult, MP sphincterotomy was performed using a needle-knife (KD-10Q-1; Olympus Medical Systems) positioned adjacent to a previously inserted guidewire. Finally, a 4 Fr NPD tube was successfully placed on the MPD ( Fig. 2 ), and SPACE was fully performed. The cytology assessed from the SPACE revealed adenocarcinoma and distal pancreatectomy with splenectomy was subsequently performed. The final histopathological diagnosis was CIS of the pancreatic duct of the pancreatic body, pTis, pN0, sM0, stage 0, with R0 resection. SPACE via NPD through the MP represents a valuable alternative for diagnosing CIS when NPD tube placement via the major papilla is difficult.

**Fig. 1:**
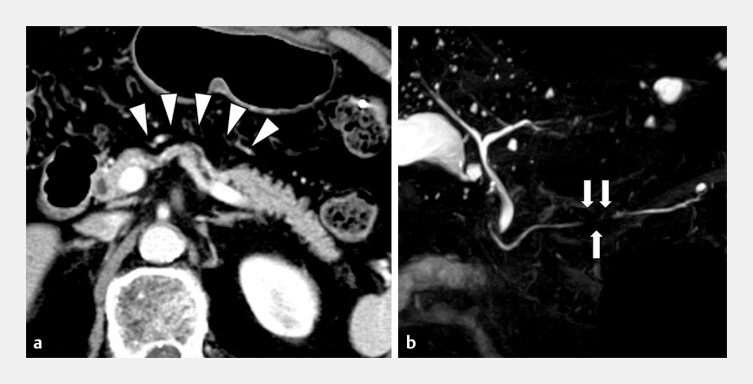
CT and MRCP images. **a** A focal pancreatic parenchymal atrophy, 30 mm in size, is present in the pancreatic body. The pancreatic mass is not visible in the CT scan (arrowhead). **b** MRCP showing the main pancreatic duct, with a slight stricture of the pancreatic body (arrow). Abbreviations: CT, computed tomography; MRCP, magnetic resonance cholangiopancreatography.

**Fig. 2:**
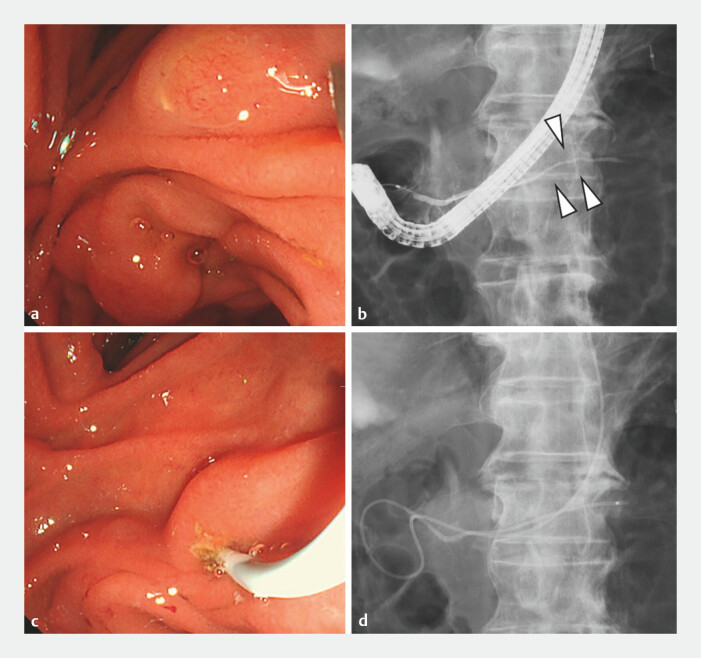
**a** The orifice of MP was detected. **b** Endoscopic retrograde pancreatography via MP shows stricture of the MPD of the pancreatic body (arrow). **c, d** A 4-Fr endoscopic naso-pancreatic drainage tube was placed in the MPD via MP. Abbreviations: MP, minor papilla; MPD, main pancreatic duct.

Endoscopy_UCTN_Code_TTT_1AR_2AD

## Notes

Endoscopy E-Videos https://eref.thieme.de/e-videos
*E-Videos* is an open access online section of the journal *Endoscopy* , reporting on interesting cases and new techniques in gastroenterological endoscopy. All papers include a high-quality video and are published with a Creative Commons CC-BY license. Endoscopy E-Videos qualify for HINARI discounts and waivers and eligibility is automatically checked during the submission process. We grant 100% waivers to articles whose corresponding authors are based in Group A countries and 50% waivers to those who are based in Group B countries as classified by Research4Life (see: https://www.research4life.org/access/eligibility/ ). This section has its own submission website at https://mc.manuscriptcentral.com/e-videos .
